# Transforming obstetric ultrasound into data science using eye tracking, voice recording, transducer motion and ultrasound video

**DOI:** 10.1038/s41598-021-92829-1

**Published:** 2021-07-08

**Authors:** Lior Drukker, Harshita Sharma, Richard Droste, Mohammad Alsharid, Pierre Chatelain, J. Alison Noble, Aris T. Papageorghiou

**Affiliations:** 1 Nuffield Department of Women’s and Reproductive Health, University of Oxford, Oxford, UK; 2 Institute of Biomedical Engineering, Department of Engineering Science, University of Oxford, Oxford, UK

**Keywords:** Clinical trial design, Diagnosis

## Abstract

Ultrasound is the primary modality for obstetric imaging and is highly sonographer dependent. Long training period, insufficient recruitment and poor retention of sonographers are among the global challenges in the expansion of ultrasound use. For the past several decades, technical advancements in clinical obstetric ultrasound scanning have largely concerned improving image quality and processing speed. By contrast, sonographers have been acquiring ultrasound images in a similar fashion for several decades. The PULSE (Perception Ultrasound by Learning Sonographer Experience) project is an interdisciplinary multi-modal imaging study aiming to offer clinical sonography insights and transform the process of obstetric ultrasound acquisition and image analysis by applying deep learning to large-scale multi-modal clinical data. A key novelty of the study is that we record full-length ultrasound video with concurrent tracking of the sonographer’s eyes, voice and the transducer while performing routine obstetric scans on pregnant women. We provide a detailed description of the novel acquisition system and illustrate how our data can be used to describe clinical ultrasound. Being able to measure different sonographer actions or model tasks will lead to a better understanding of several topics including how to effectively train new sonographers, monitor the learning progress, and enhance the scanning workflow of experts.

## Introduction

There are few population screening programs that rely on imaging as the primary screening modality—mammography, aortic aneurysm screening and screening during pregnancy are three^1^. Of these, worldwide, obstetric ultrasound is by far the most used. Ultrasound is a relatively low-cost medical imaging modality, compared to X-ray, CT and MRI, is convenient and painless, does not use ionizing radiation, yields immediate results, and is widely considered to be safe^2^. In the past decade and by virtue of advancement in the understanding of fetal functional anatomy, standardization of scanning guidelines, clinical diagnosis, and technical developments of image acquisition equipment, the quality of obstetric ultrasound has improved. In particular, advances in ultrasound physics, materials science, electronics, and computational power have collectively resulted in improvements in image quality and capabilities: higher spatial and temporal resolution as well as increased signal-to-noise ratio, minimization of artifacts, and three-dimensional (3D) rendering^3–9^.

**Figure 1:**
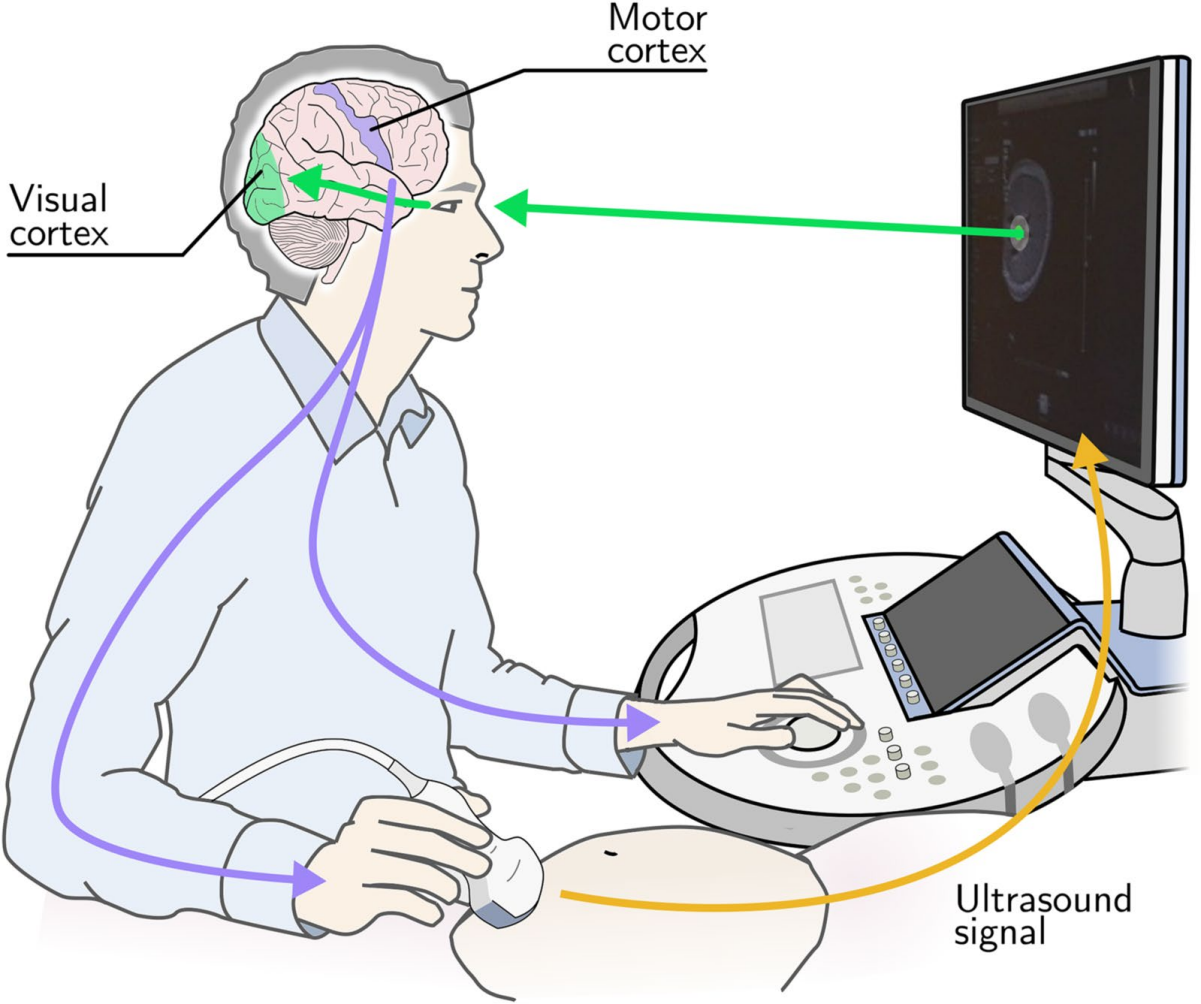
"Sonographer loop" is the process ultrasound sonographers undertake while acquiring standard planes: the sonographer gazes at the ultrasound screen, manipulates the hand to adjusts the transducer position which changes the displayed image, and back to sonographer looking at the monitor. Figure created using Inkscape version 0.92 (https://inkscape.org/).

Unlike these achievements, the process of ultrasound scanning, the technique of finding standard anatomical planes of the fetus to allow their diagnostic examination, remains relatively unchanged. Routine obstetric ultrasound scans are performed by a sonographer sitting or standing next to a pregnant woman lying down, manipulating a transducer and adjusting the machine settings, following a defined protocol, in order to acquire a series of two-dimensional standard imaging planes, observed on the screen of the ultrasound machine. This process can be thought of as the “sonographer loop”: the sonographer moves the transducer looking for a standard plane, receives real-time visual feedback of the video on the display screen and constantly moves their hand to adjust the transducer position, which changes the displayed video (Fig. 1).

Deep learning is a highly sophisticated pattern recognition methodology, well suited, in theory, to recognizing image appearance characteristics associated with diagnostic criteria. This is a key reason why deep learning is currently having an impact on radiology for automation of reading of computed tomography (CT), magnetic resonance imaging (MRI), X-ray and optical imaging^10–14^. Ultrasound imaging is unique in the level of high skill required to acquire *and* interpret imaging “on-the-fly”. Obtaining a high‐quality and informative diagnostic ultrasound image requires substantial expertise, a skill traditionally acquired over several years. In addition, in the case of obstetric ultrasound, image quality depends on maternal habitus, fetal movements and fetal position^7^. To improve sonographer accuracy, efficiency, and productivity, we need to further our understanding of the interaction between the sonographer, transducer and live image^15–18^.

The PULSE (Perception Ultrasound by Learning Sonographer Experience) project is an interdisciplinary effort that aims to understand the entire obstetric imaging scanning process as a data science problem. For this purpose, we have developed a custom-built dedicated multi-modality ultrasound system that acquires the ultrasound scan and logs the sonographer actions by simultaneously recording full-length ultrasound video scans, eye-tracking data, transducer motion data, and audio of the sonographer speaking. Our system is deployed in a tertiary hospital clinic to capture data during routine obstetric ultrasound scanning for women attending the clinic for first, second and third trimester scans.

The aims of the paper were, first, to outline the PULSE methodology for the acquisition of data on sonographer perceptions and actions. To the best of our knowledge, the system, and dataset are unique. Second, we describe some of the deep learning image analysis models that have been built using data from the multiple perceptual cues. Third, we present lessons leant from the new field of ultrasound big data science. Fourth, we discuss some future technical research directions and possible new clinical applications of the emerging deep learning-based technology.

## Methods

In this prospective data collection study, we record anonymized full-length obstetric ultrasound scan videos while tracking the actions of the sonographer. In the current paper we describe the data acquisition process of the PULSE study, explain how we interpret the multimodal data, and present preliminary clinically meaningful analyses.

### Routine care settings

Recruitment began in May 2018. During the study period, as part of standard care, all women booked for maternity care in Oxfordshire, in the United Kingdom, are offered three routine ultrasound scans during pregnancy: in the first trimester at 11–13+6 weeks, which includes assessment for viability, gestational age assessment and an offer of aneuploidy screening by measurement of the nuchal translucency; a 20-week anomaly scan; and assessment of fetal growth at 36 weeks^19,20^. Ultrasound examinations are carried out by accredited sonographers, supervised trainees or fetal medicine doctors using standard ultrasound equipment. For quality assurance purposes, the stored images and the reliability of measurements are regularly assessed by a senior accredited sonographer using established quality criteria^21^.

If an abnormality is suspected during a routine scan, the pregnant woman is referred for further evaluation at the Fetal Medicine Unit, Oxford University Hospitals National Health Services (NHS) Foundation Trust. Such scans are not included in the current study.

### Pregnant women as participants

Pregnant women attending routine obstetric scans are invited to participate in the study by agreeing to have their ultrasound scan recorded by video. The inclusion criteria are age > 18 years old and the ability to provide verbal and written informed consent in English. Multiple gestations are not excluded. Following consent, each pregnant woman is given a unique study participant number to allow the anonymization of data.

The routine ultrasound scan is carried out without any modifications for the purpose of this study. Hence, ultrasound scans are performed according to national and unit guidelines. As for all women, scan results are given to the woman after the ultrasound examination and further management is performed according to national and local protocols by the appropriate healthcare professional. Women are not required to attend any follow-up or subsequent research scans. Women who consent to participate, but subsequently request to be withdrawn from the study, are excluded from the analysis.

### Sonographers as participants

Participating sonographers are accredited sonographers, trainees supervised by accredited sonographers, or fetal medicine doctors. For the purpose of this study, we refer to all ultrasound operators, sonographers and fetal medicine doctors, as sonographers. The sonographer inclusion criteria are practicing obstetric ultrasound, and willing and able to give informed consent in English for participation in the study. Following written informed consent, each sonographer is assigned a unique study number. All participating sonographers are given an introduction about the purpose and aims of the study, followed by specific individual training as required.

Sonographers are requested to maintain the routine scanning procedures and are not required to alter their practice for the purpose of the study.

### Data collection

#### Ultrasound system

All scans are performed using a commercial General Electric (GE) Healthcare Voluson E8 or E10 (Zipf, Austria) ultrasound machines equipped with standard curvilinear (C2-9-D, C1-6-D, C1-5-D) and 3D/4D transducers (RAB6-D, RC6M). The system is equipped with customized additions for recording scans, eye-tracking and transducer motion. Tracking of the vaginal transducer is not part of the study.

#### Video recording

The secondary video output from the ultrasound machine is connected to a computer equipped with a video grabbing card (DVI2PCIe, epiphany video, Palo Alto, California) and purpose-built software to ensure real-time anonymization of the video. Hence, the saved videos include no personal details. Full-length ultrasound scans are recorded using the ultrasound machine full high-definition (HD) resolution (1920 × 1080 pixels) at 30 frames per second. Video files are recorded using lossless compression.

#### Eye tracking

Eye tracking is achieved using a remote eye-tracker (Tobii Eyetracking Eye Tracker 4C, Danderyd, Sweden) mounted below the ultrasound machine display monitor. For each ultrasound frame, we record the exact point of sonographer gaze. The accuracy of the eye-tracking method in our setting was previously validated^22^. Sonographers do not have any visual or other signal to know that the eye-tracking device is functioning.

#### Transducer tracking

**Figure 2:**
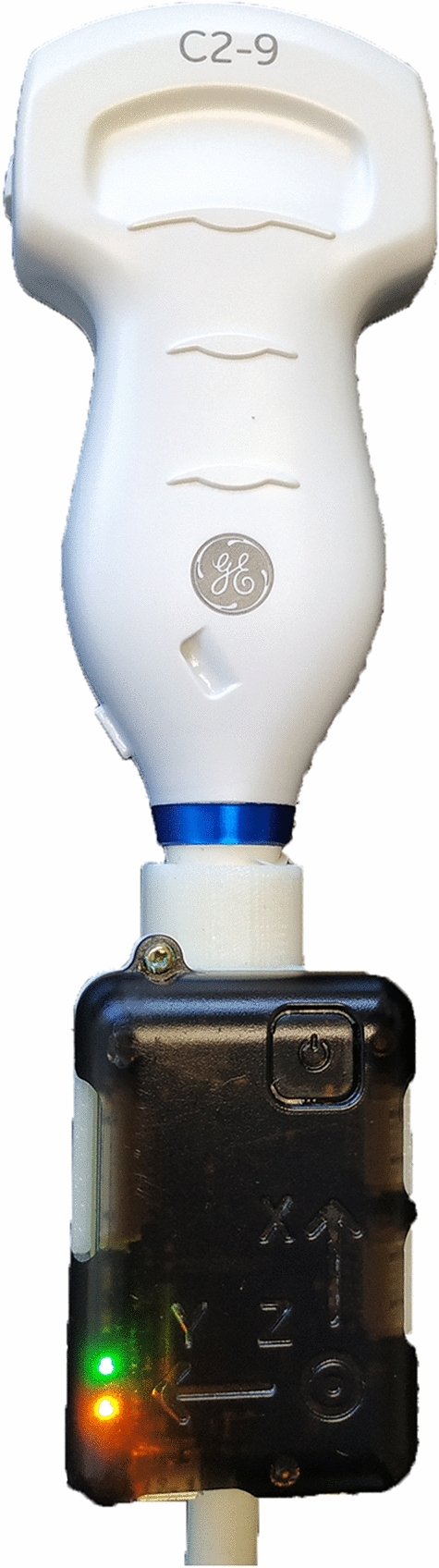
Inertial Measurement Unit (IMU) device attached to a curvilinear ultrasound transducer, acting as the sonographer hand tracking device. Figure created using Microsoft Office version 365 (https://office.microsoft.com/).

Transducer motion tracking is achieved using an Inertial Measurement Unit (IMU), a motion sensor, (NGIMU, X-IO Technologies, Bristol, UK) attached to the ultrasound transducer (Fig. 2). The three-axis accelerometer information is recorded at 100 Hz.

#### Audio recording

Sonographer voice recording is carried out using two microphones (PCC160, Crown HARMAN, Northridge, California). One microphone is located in proximity to the operator, next to the ultrasound machine display screen, to best capture the operator’s voice. The second microphone is located away from the operator, next to the pregnant woman and any accompanying persons. This setup allows to isolate the sonographer’s voice from that of others present in the scanning room. Differentiation of the operator’s voice from that of others is carried out using the VoxSort Diarization software (Integrated Wave Technologies) and transcription is performed using the ELAN^23^ annotation tool.

#### Sonographer interaction with ultrasound machine

Ultrasound machine keystrokes and cursor movement result in visual cues. Detection of the bidirectional sonographer-ultrasound machine communication is carried out with a custom-designed software. The software automatically processes the ultrasound video to determine sonographer manipulation of the ultrasound controls and machine-displayed values, allowing detection of events like “freeze”, “save”, or “clip save”, and machine-displayed values such as thermal safety indices, or measurement values. The purpose-built software, which performs a frame-by-frame analysis, was implemented in Python (http://www.python.org, version 3.7.0) using OpenCV (http://www.opencv.org, version 3.4) and Tesseract (http://www.github.com/tesseract-ocr, version 3.05) libraries. Extracting the machine parameters displayed in the graphical user interface was achieved through pattern recognition and optical character recognition (OCR).

### Sample size

Owing to the observational nature of the study, calculating a meaningful clinical sample size is challenging. For machine learning, it has been previously suggested that “the more the better”^24^. Based on logistical and clinical considerations, including an aim to achieve an adequate sample across different sonographers and across fetal gestational age, we prospectively chose to recruit a total of 3000 routine scans, with up to 20 sonographers performing these scans, to provide approximately 1000 scans for each gestational age group.

### Data analysis

**Figure 3:**
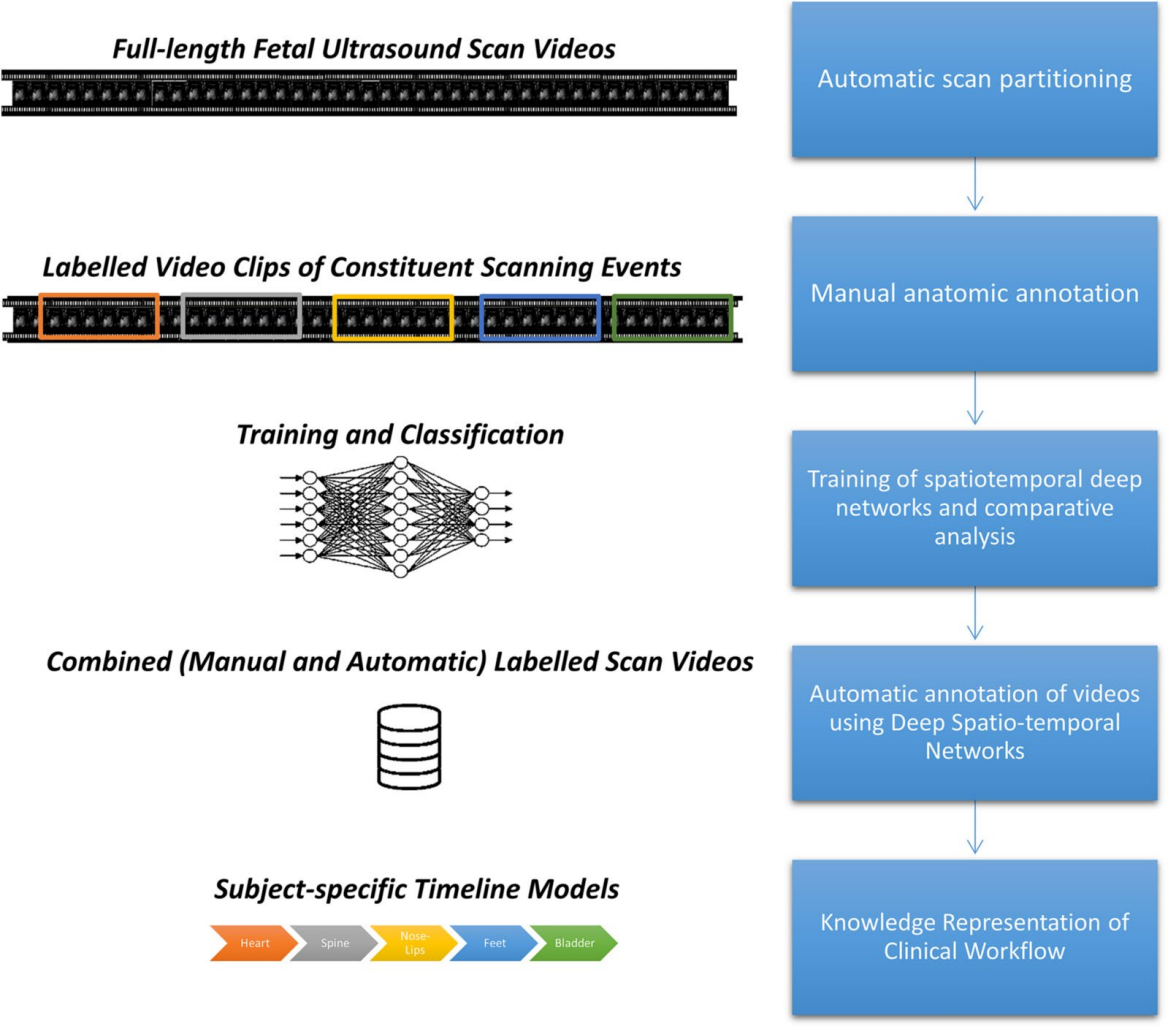
Outline of the clinical workflow analysis pipeline. Stepwise approach to second-trimester workflow analysis: (1) partitioning—automatically extraction of 5-s clips from the videos that represent important events; (2) manual labeling of the training dataset; (3) training a spatiotemporal deep network; (4) automatic labelling of the entire dataset; and (5) analysis of second-trimester scans as a sequence of organs being scanned. Figure created using Microsoft Office version 365 (https://office.microsoft.com/).
  *Episodes of interest*: For each scan video, the customized software detected episodes (periods of time) where a defined screen area was being looked at (such as the "measurement box" or the “bioeffect box”). The software detected uninterrupted fixations, defined as uninterrupted sonographer gaze toward the area of interest, lasting ≥100ms. If the fixation is interrupted, this was considered as a single episode of eye fixation if this interruption was 400 ms or less; or as a separate fixation if it is more than 400 ms^28,29^. We verified this choice of threshold by randomly looking at more than 50 detected fixations and making sure that the threshold resulted in no false positives.

The data collected present holistic and very rich information about obstetric sonography. In order to analyze the data, the following strategies were used:

1. Second-trimester workflow analysis using video understanding (Fig. 3). Since the second-trimester anomaly scan represents the most widely conducted obstetric ultrasound scan, we chose to analyze the workflow of these scans. *Partitioning*—initially, we automatically extracted 5-s clips from the videos that represent important events by automatic detection of video freeze, image save, or clip save. We defined these as important scan events because sonographers freeze the screen when concentrating on an acquisition and image/clip save is carried out when the sonographer is satisfied with the acquisition displayed on the monitor.
  *Labeling*—The very large number of 5-s clips makes it impractical to manually label all of them. Therefore, we developed a deep learning model to automate this process. We first carried out manual labeling of approximately 20% of the available video clips according to the anatomy/organ/standard plane: head–brain, face (axial, coronal, or sagittal), arms, hands, situs, thorax–heart, abdomen, umbilical cord abdominal insertion, genitalia, bladder, legs, femur, feet, spine, kidneys, full-body fetal sagittal view, placenta-amniotic fluid, maternal anatomy (like uterine artery), 3D/4D mode, mixed, and unidentified. Thereafter, we labeled approximately 300 s-trimester scans, using machine learning method^25,26^. In brief, we developed and trained deep spatiotemporal networks, after an exploratory analysis of several deep architectures such as 2D ConvNets, 3D ConvNets, Recurrent Neural Networks, model fusion and initialization techniques^25^. We comparatively evaluated the optimally-learned spatiotemporal deep neural network, used it to classify sequential events in unseen full-length ultrasound scan videos, and temporally regularized the predicted result.
  At the final step, each second-trimester scan was represented as a continuous line of fetal standard plane (anatomy/organ) versus time. This chosen layout allows easy visualization and interpretation of the type, duration and order of the anatomy being evaluated.
2. Motion: The inertial measurement unit (IMU), which is attached to the ultrasound transducer, was used to record the rotational rate and linear acceleration for each axis of the sensor. The rotational rate fused with additional sensory information (gravity, magnetic field) is fed into an attitude and heading reference system (AHRS) that computes the absolute orientation in 3D space. We analyzed the transducer movement to compute the three-dimensional orientation and linear acceleration of the transducer throughout full-length scans.
3. Natural language of ultrasound: audio recordings were transcribed into text to build machine learning models for image captioning. These textual captions allowed studying the language sonographers use while performing a scan. Breakdown of the speech from a representative sample of scans determined that the distribution of adjectives, determiners, nouns, and verbs was 12.7%, 22.2%, 28.0%, and 16.0%, respectively^27^. The remaining 21.1% were prepositions, pronouns, adverbs, and other parts-of-speech.
4. Eye-tracking: Using eye tracking we were able to determine the objects that a sonographer looks at. For instance, in the video clip 1 (Video S1), initially, it appears that the fetal profile is being examined; but the second part of the clip shows, by displaying the eye-tracking point on the video, that in fact the fetal brain is being studied (note that the green dot is added to the video to facilitate the presentation of our findings, and is not visible to a sonographer in the study).

### Ethics approval

Ethics approval was granted by the West of Scotland Research Ethics Service, UK Research Ethics Committee (Reference 18/WS/0051). All methods were carried out in accordance with relevant guidelines and regulations. Written informed consent was obtained from all participants.

## Results

### Workflow analysis

**Figure 4:**
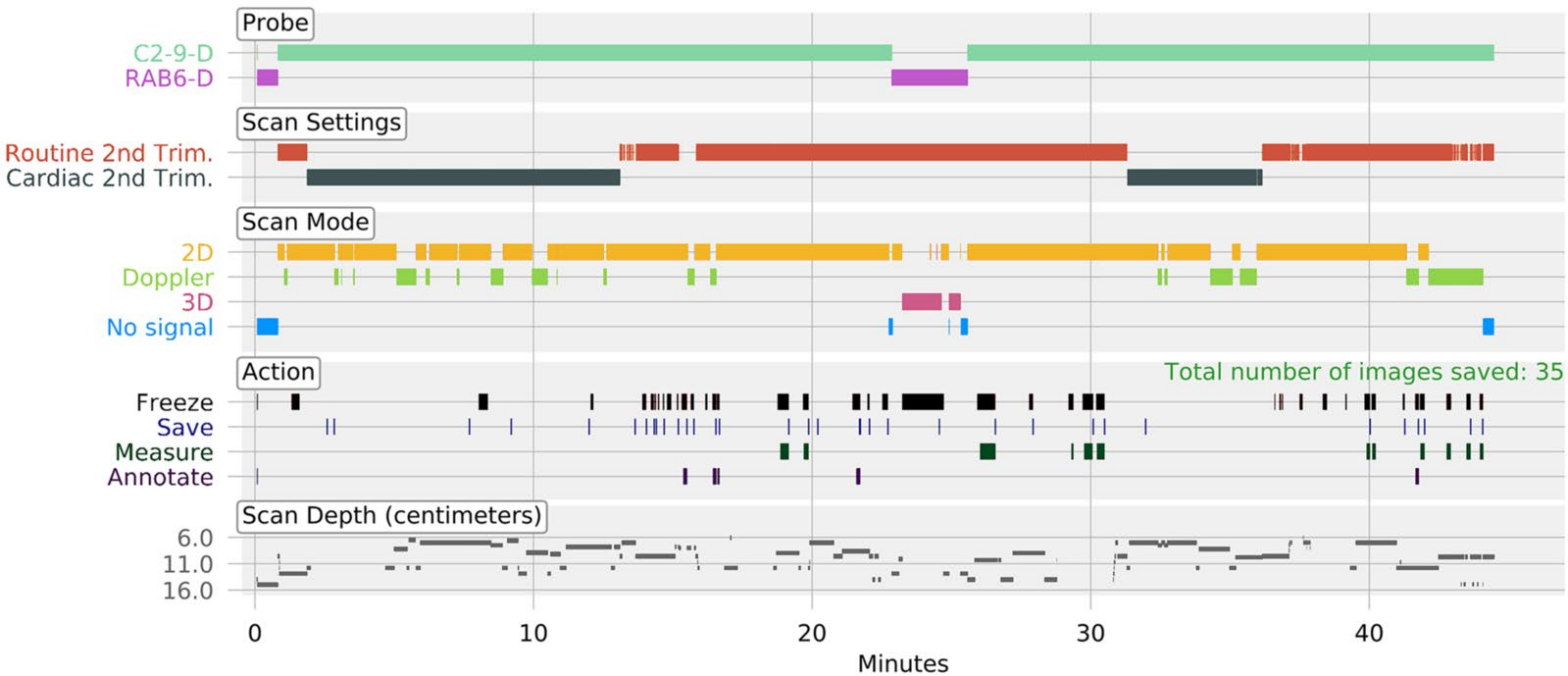
Anomaly scan machine parameters and sonographer actions according to time in one representative anomaly scan. A representative second-trimester anomaly scan. The overall duration of the scan was approximately 45 min, of which the majority of time was live scanning with the C2-9D probe. A large proportion of the scan was dedicated toward cardiac scanning and 35 episodes of image same were detected. Figure created using Python's Matplotlib library^53^ and Microsoft Office version 365 (https://office.microsoft.com/).

A total of 341 full-length second trimester anomaly scans, performed by ten sonographers, with an average duration of 36.2 ± 11.6 min per scan, were available for analysis (65 K frames/anomaly scan), representing a total of approximately 205 h of video. Figure 4 shows the automatically extracted machine settings and sonographer actions in one typical full-length anomaly scan. In this representative anomaly scan, the overall duration was 44 min, of which 77% was in live-scanning, and a total of 35 images and clips were saved.

**Figure 5:**
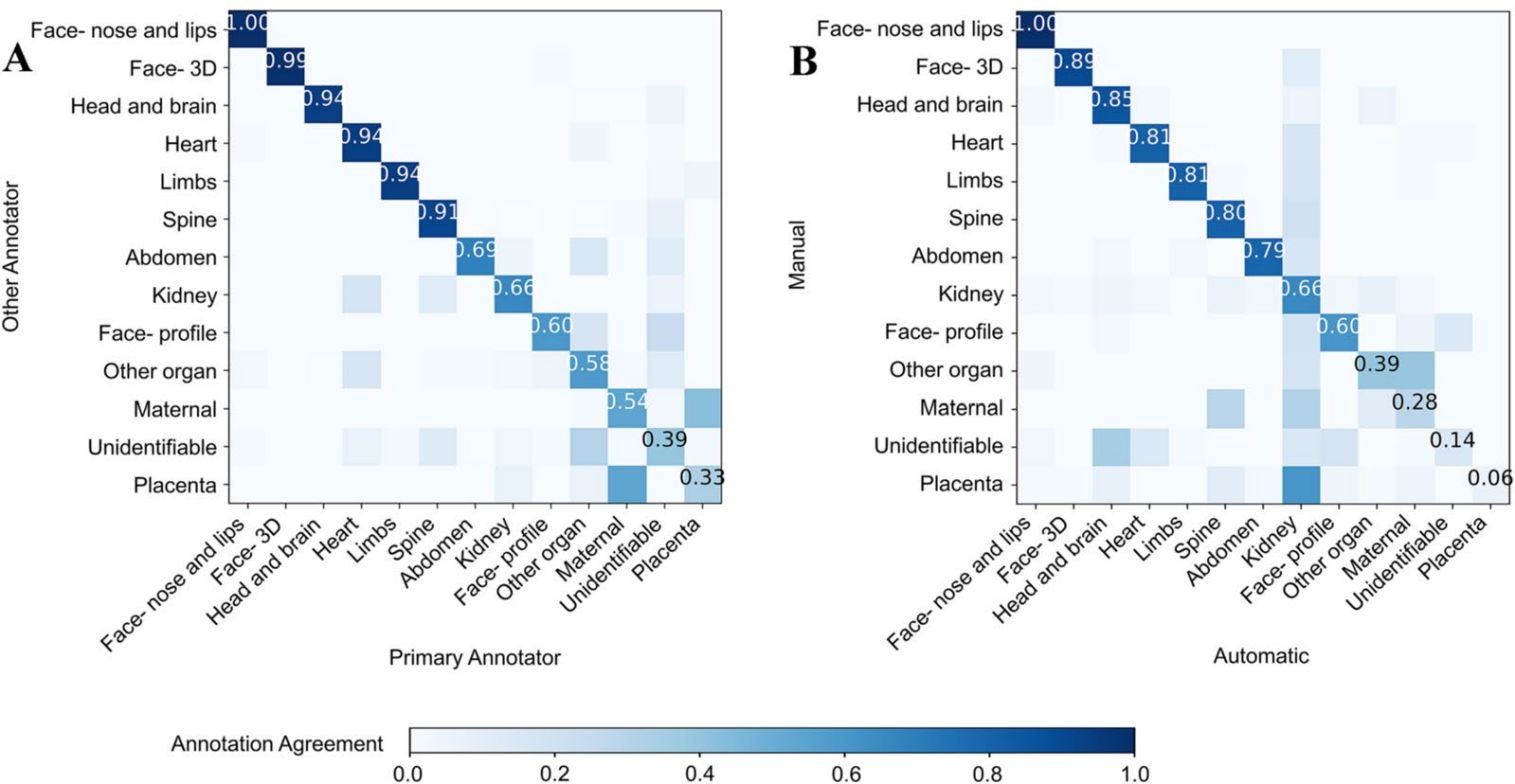
Confusion matrices for the comparison of scans labeling. (**A**) Scans labeled by different human annotators and (**B**) Scans manually labeled vs. those automatically labeled by a deep neural network. Figure created using the Python programming language (Python Software Foundation, https://www.python.org/).

Out of the 341 scans, 62 anomaly scans were randomly selected and manually labeled (annotated) as the training dataset by a clinical obstetric ultrasound expert and seven image analysis scientists with knowledge of obstetric scanning. A high inter-annotator agreement (76.1%) was found between the annotators and a confusion matrix comparing the primary annotator and other annotators is displayed in Fig. 5A. We note that for a few labels, representing structures often seen together, the confusion was high—for example hands and face—as the fetal hands are often in proximity to the face, making both visible in the same image (Fig. 5A). To train a deep neural network, the 12 most commonly used labels were selected, representing 88% of the 5-s clips (Table 1). A supervised deep learning network architecture for automatic temporal semantic labelling performed the labelling of the 279 remaining un-labeled full-length anomaly scans. A sample of 28 scans (10%) from the automatically labelled dataset was randomly selected and manually labeled. Figure 5B presents a confusion matrix comparing the agreement between manual and automatic labeling which overall was found to be 76.4%. Statistical analysis of the manual and automatically labelled scans showed a high Pearson’s correlation ρ = 0.98 (p < 0.0001). Also, there was relatively high disagreement between labels that often display more than one structure at the same time: placenta and maternal structures as well as kidneys and abdomen (Fig. 5B).

**Table 1:** Prevalence of the 12 most common anatomical labels in 62 full-length second-trimester anomaly scans.

Anatomical label	Prevalence (%)
Thorax–heart	19.6
Head–brain	11.5
3D/4D mode	11.2
Unidentified	11.0
Spine	6.1
Abdomen	5.5
Maternal anatomy	5.5
Coronal face	4.4
Placenta-amniotic fluid	4.2
Sagittal face (profile)	3.3
Kidneys	2.9
Femur	2.7

**Figure 6:**
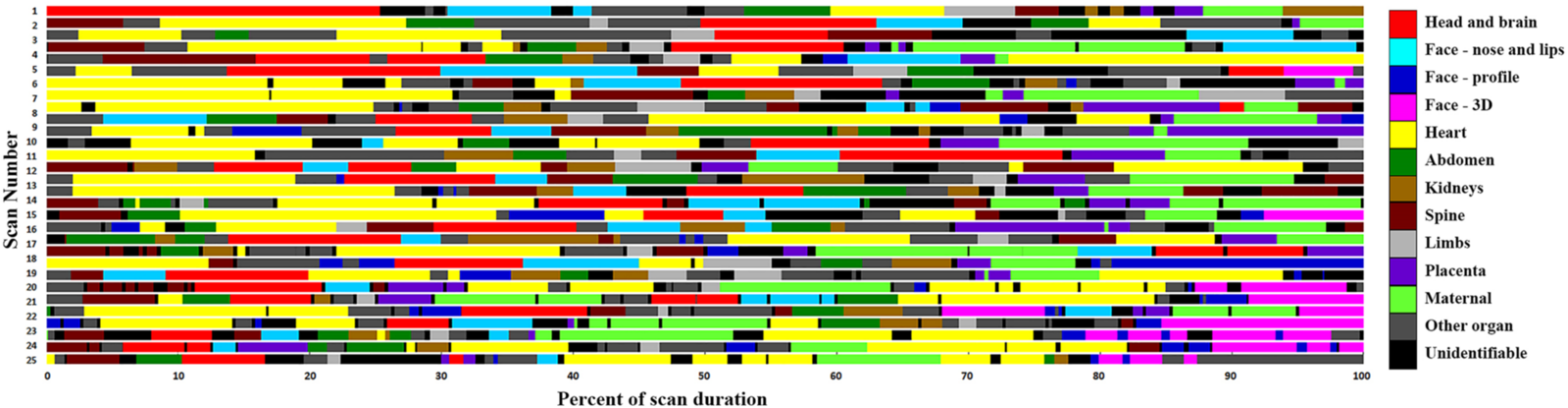
Anatomic plane being evaluated according to time (normalized to percent) in 25 representative anomaly scans. Figure created using MATLAB version 9.6 (https://www.mathworks.com/).

With all scans labelled, we ended up with 1,158,782 labelled video frames. This allowed the anomaly scans to be visualized by organ as a function of time (25 representative scans are shown in Fig. 6). From the time-based visualizations, on average, 21.5% (20.4–22.6%) of the scan length was dedicated to cardiac imaging, and 11.4% (10.8–12.0%) to the fetal head and brain (Table 2). It is also evident that on several scans, such as scan #25, the sonographer dedicated multiple episodes to scan the fetal heart.

**Table 2:** Proportion of scan duration dedicated toward each of the 12 most common anatomical labels in 341 full-length second-trimester anomaly scans.

Anatomical label	Average (95% CI) duration dedicated to anatomy
Thorax–heart	21.5% (20.4–22.6%)
Head–brain	11.4% (10.8–12.0%)
3D/4D mode	3.1% (2.6–3.6%)
Unidentified	17.7% (16.7–18.7%)
Spine	8.8% (8.0–9.5%)
Abdomen	5.9% (5.4–6.4%)
Maternal anatomy	10.5% (9.8–11.1%)
Coronal face	4.1% (3.7–4.4%)
Placenta-amniotic fluid	4.3% (3.9–4.8%)
Sagittal face (profile)	5.2% (4.7–5.8%)
Kidneys	1.6% (1.3–1.8%)
Femur	3.0% (2.7–3.3%)
Other organ	3.1% (2.4–3.8%)

Data is percent (95% confidence interval).

### Transducer motion

**Figure 7:**
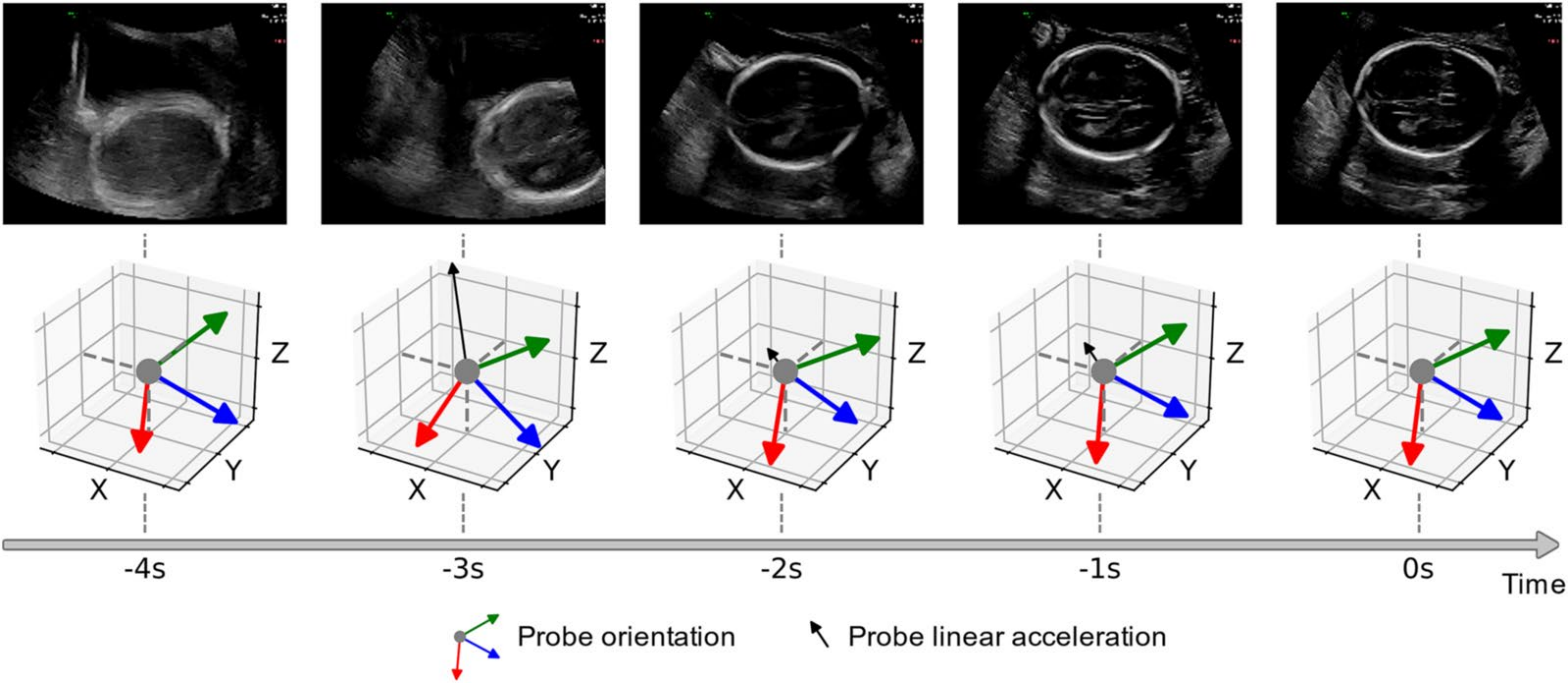
Example of transducer motion data. The figure displays the transducer motion data during the acquisition of the trans-ventricular (TV) standard biometry head and brain plane. The first row shows the ultrasound machine displayed frames (in 1-s intervals) before freezing (time = 0 s). The second row shows the corresponding transducer orientation and acceleration vector for each frame. Figure created using Python's Matplotlib library^53^ and Inkscape version 0.92 (https://inkscape.org/).

Figure 7 shows frames from a representative 5-s acquisition of the head trans-ventricular standard biometry plane and corresponding ultrasound transducer motion information in 1-s intervals. It is evident that the sonographer continuously manipulated the transducer position in order to acquire the desired imaging plane. The example shows the link between transducer orientation and linear acceleration and the ultrasound image. This motion information revealed how the sonographer arrived at the desired view, which is not implicitly seen from the video data by itself.

### Image captioning

**Figure 8:**
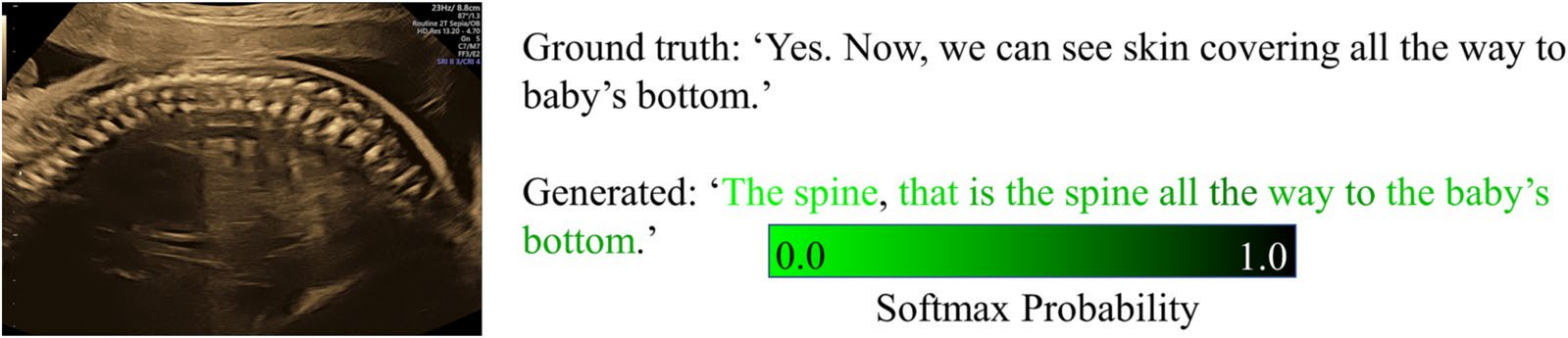
Image captioning: the ground truth and a generated caption are shown for the fetal spine. The darker the green color, the higher probability of the associated with a generated word (Softmax probability). Figure created using Python's Matplotlib library^53^ and Microsoft Office version 365 (https://office.microsoft.com/).

We empirically observe that operators use sentences that are up to 83 words long when describing ultrasound video with a vocabulary consisting of approximately 344 unique words. The small number of unique words in this sonographer vocabulary demonstrates that sonographers tend to speak in a very particular way to describe the content of ultrasound scans. Using this real-world sonographer vocabularies, we were able to build image captioning models to generate relevant captions for anatomical structures of interest that appear in routine fetal ultrasound scans^27^. Figure 8 compares a caption spoken by a sonographer with one generated by our automatic natural language processing (NLP) based image captioning method that models the vocabulary commonly used by operators. It is notable that the generated caption is similar to the operator spoken word in terms of sonographer linguistic style.

### Safety of ultrasound

The Thermal Index is an indicator of the risk of tissue heating, displayed by standard ultrasound machines. To ensure safety, ultrasound sonographers are required to adhere to recommended ultrasound thermal safety indices exposure times^30^. Using PULSE video and eye tracking data we can study how well sonographers adhere to the recommendations in practice. Specifically, software was implemented to automatically monitor the values displayed and gaze toward the “bioeffect box” displayed by the ultrasound machine. 17 sonographers performed 178, 216, and 243 ultrasound scans at the first, second and third trimesters, respectively. Gaze tracking showed that the displayed “bioeffect box” was looked at in only 27 of the 637 routine scans (4.2%). Despite the infrequent assessment of the thermal safety indices, the machine presets allowed for the recommended thermal safety indices to be kept during all routine scans and using the different ultrasound modes: B-mode, Color/Power Doppler, and Pulsed Wave Doppler^31^.

### Bias in biometric measurements

Fetal biometry is an essential part of the majority of pregnancy scans. It is the focus of the examination in the third trimester, and variability in fetal measurements is also higher at this gestation. Therefore, we analyzed a total of 272 third-trimester scans performed by 16 sonographers with associated gaze data that were available for analysis of the standard biometric measurements. During these scans, we automatically identified 1409 biometric measurements: 354 head circumference, 703 abdominal circumference, and 352 femur length. During biometry, ultrasound machines display the “measurement box” that displays the measurement result. Eye-tracking demonstrated that sonographers look at the ultrasound machine displayed values in > 90% of standard fetal biometric measurements. This bias causes the sonographers to "correct" the measurement by adjusting the caliper placement toward the expected measurement for the actual gestational age^32^.

## Discussion

In this paper, we demonstrate that ultrasound data science can offer fresh new clinical insights to understand routine real-world obstetric ultrasound screening imaging. We explain how, using multi-modal scanning data, we can build deep learning models of clinical sonography and image analysis. To be able to describe how expert sonographers perform a diagnostic study of a subject, we present a novel multi-cue data capture system that records the scan concurrently with tracking the actions of the sonographer, including interaction with the machine, sonographer eye and transducer movements. By measuring different sonographer actions or tasks, we further aim to understand better several topics such as how to effectively and efficiently initially train sonographers, monitor learning progress, and enhance scanning workflow. Our data can also help design deep learning applications for assistive automation technology as described elsewhere^33–36^.

Understanding sonography in quantitative detail allows us to show clinically meaningful examples such as workflow analysis, image captioning, bias identification and thermal safety indices monitoring. The workflow of obstetric ultrasound is a serial task of non-ordered standard planes acquisition and interpretation. For each standard plane, the process starts with the detection of the main anatomical region, fine-tuning to the target structure followed by an optimal acquisition in the correct plane, verifying normal appearance, measuring as required and ultimately storing. We have presented a novel system that records an entire ultrasound scan and partitions it according to the standard plane/organ being temporally studied, which results in a quantitative visualization of plane/organ vs time. This allows us to study ultrasound workflow as information science. For example, we observe that sonographers spend a large proportion of the scan undertaking fetal cardiac assessment, yet there is a large variation between sonographers. This could potentially mean that a specific sonographer may be struggling with this part of the scan and might, therefore, benefit from further training. Thus, analysis and quantification of ultrasound scans can allow monitoring of learning, identification of weaknesses, auditing, and verifying scan completeness. Ultimately, this should improve the cost-effectiveness of scans. Previous work in ultrasound learning and workflow in non-obstetric ultrasound fields has resulted in the creation of automated algorithms to aid transducer guidance, automatic scanning, and image analysis^37,38^. In obstetrics, real-time automatic identification of standard planes may aid diagnosis and quality assurance^33,36,39–46^.

Eye-tracking has revealed important sonographer behaviors, including the tendency to bias fetal biometry measurements. Such operator bias is important for research and clinical settings and require further prospective assessment to understand its source and impact. Another important lesson learned from eye-tracking is that sonographers do not adequately monitor ultrasound thermal safety indices. This means that algorithms should be specifically designed to adhere to safety recommendations and not just “learn” how to scan from sonographers. Additionally, automated assistance to sonographers in monitoring of thermal safety indices should be implemented; ultrasound machines should not just report the safety parameters, but also be designed to actively avoid exceeding exposure times or alert the sonographer if the safety parameters are breeched.

### Planned analyses

In our study, in addition to ultrasound video, we store real-time information of transducer motion and eye-tracking. This additional sensory data is particularly interesting as it has the potential to improve machine learning algorithms for obstetric ultrasound related tasks using expert knowledge that is not available from ultrasound video alone.

Ultrasound transducer guidance is already available commercially for adult echocardiography^47^, and image-based guidance solutions have been proposed for instance for ultrasound-guided regional anesthesia^37^. The transducer tracking data captured in this study will lead to an understanding of how to best maneuver a transducer to acquire the appropriate planes, and in turn, allow the development of “guidance” of the transducer to the correct scan plane. Due to the highly variable anatomy that is dependent on fetal position, acquiring these skills usually requires years of training. An algorithm that guides the transducer to the correct position in real-time potentially has an enormous advantage over the current one-on-one teaching that includes a lot of human trial-and-error. An early report on our work on this topic is described in^48^.

The tasks captured by sonographers during a routine fetal anomaly ultrasound scan are characterized by properties such as their order and their duration, which are not strictly constrained in the scanning protocol. Using deep learning on ultrasound video, we comprehensively analyze and quantify sonographer clinical workflow in terms of the type, duration and sequence of the tasks constituting a full-length scan. Next, we plan to attempt improving the accuracy of the sonographer and automatically generated image description. Thereafter, we aim to evaluate the utility of the sonographer tracking in improving the workflow assessment model.

### Research and clinical potential

In this study, we explore how the latest ideas from deep learning can be combined with clinical sonography big data to develop deep models and understanding to inform the development of the next generation of ultrasound imaging capabilities and make medical ultrasound more accessible to non-expert clinical professionals.

Despite half of a century of ultrasound usage, most advances have been in sensors and electronic systems, leaving the sonographer behavior field largely unexplored. We attempt to bridge the gap between an ultrasound device and the user by employing machine learning solutions that embed clinical expert knowledge to add interpretation power. The hope is that taking this approach may provide a major step towards making ultrasound a more accessible technology to less-expert users across the world. It is widely known that a shortage of sonographers and radiologists are a major bottleneck to ultrasound access worldwide^49,50^ and that ultrasound sonographers are at risk for burn-out, and musculoskeletal injuries that potentially limit their professional work. Sonographers who have been clinically active for decades probably scan in a manner that poses minimal risk for injury. By capturing the actions of such experts, we may determine important characteristics of scanning that may prevent sonographer injury.

### Strengths

The novel design that allows us to understand human perceptual information by recording the sonographer eye and transducer movements during real-life scans, while recording the resulting ultrasound video, are major strengths of the study. We have already shown robust constant findings from capture and analysis of multiple scans and sonographer. This includes verification of the ability to detect important scan events, to extract data from the ultrasound image, and to accurately identify the sonographer gaze. Also, in light of emerging adoption of artificial intelligence in radiology^51,52^, the ability to implement related methods into obstetric ultrasound is likely to progress the field of clinical obstetric sonography.

### Limitations

Our study is not without limitations. Mainly, we present the study design and preliminary findings that require further in-the-field clinical validation. Also, before such an algorithm could be implemented in clinical practice, we aim to improve the inter-annotator and manual to automatic agreement accuracy. Currently, the relatively high agreement allows interpretation of data, but an algorithm to fit clinical settings, the agreement should be improved. Additionally, the limited number of sonographers in our ultrasound unit make it difficult to convincingly assess the differences between novice and expert sonographers. However, the need for thorough clinical validation is expected with any novel idea and we were able to show the preliminary findings are easily interpretable.

## Conclusion

In conclusion, we have applied the latest ideas from deep learning to a vast amount of real-world clinical data from multiple perceptual cues, to describe and understand how sonographers scan and ultrasound images are acquired. We are currently using the derived deep learning models to design new assistive tools for ultrasound acquisition and image interpretation. These tools aim to maximize the diagnostic capabilities of ultrasound as well as improve the accuracy and reproducibility of sonography to contribute to advancing its use in the developed and developing world.

## Supplementary Information

Supplementary Video S1. Routine ultrasound scan with eye-tracking data showing that the addition of tracking emphasizes the part being studied by the sonographer. In this clip, without eye-tracking, it seems that the fetal profile is being assessed. However, with eye-tracking, it is evident that the brain is being studied on a sagittal plane. Eye-tracking data is represented by a green dot that is invisible to the sonographer. Routine ultrasound scan with eye-tracking data showing that the addition of tracking emphasizes the part being studied by the sonographer. In this clip, without eye-tracking, it seems that the fetal profile is being assessed. However, with eye-tracking, it is evident that the brain is being studied on a sagittal plane. Eye-tracking data is represented by a green dot that is invisible to the sonographer.

## Data Availability

The datasets analysed during the current study are not publicly available due to patient data governance policy. Analyses performed during the current study are available from the corresponding author on reasonable request.
